# Epidemic-induced changes in nursing students’ professional identity: a qualitative investigation

**DOI:** 10.3389/fpubh.2025.1565212

**Published:** 2025-05-02

**Authors:** Huiting Zhang, Wenhao He, Dan Cao, Jiali Liu, Yongfeng Yang, Xiaoli Zhu, Mengxiao Jiang

**Affiliations:** ^1^Department of Breast Cancer, State Key Laboratory of Oncology in South China, Guangdong Provincial Clinical Research Center for Cancer, Sun Yat-sen University Cancer Center, Guangzhou, China; ^2^Department of Orthopedic Oncology, State Key Laboratory of Oncology in South China, Guangdong Provincial Clinical Research Center for Cancer, Sun Yat-sen University Cancer Center, Guangzhou, China; ^3^Department of Radiotherapy, State Key Laboratory of Oncology in South China, Guangdong Provincial Clinical Research Center for Cancer, Sun Yat-sen University Cancer Center, Guangzhou, China; ^4^Department of Anesthesiology, State Key Laboratory of Oncology in South China, Guangdong Provincial Clinical Research Center for Cancer, Sun Yat-sen University Cancer Center, Guangzhou, China; ^5^Department of Nursing,Guangzhou Institute of Cancer Research, The Affiliated Cancer Hospital, Guangzhou Medical University, Guangzhou, China; ^6^Department of Urology, State Key Laboratory of Oncology in South China, Guangdong Provincial Clinical Research Centeer for Cancer, Sun Yat-sen University Cancer Center, Guangzhou, China

**Keywords:** COVID-19, nursing students, professional identity, qualitative research, socialization theory

## Abstract

**Objective:**

To explore the impact and mechanisms of COVID-19 on the professional identity of nursing students, providing theoretical and practical insights to inform nursing education reform.

**Method:**

This study employed Colaizzi’s descriptive phenomenological method to explore nursing students’ experiences. Semi-structured interviews were conducted, and data collection continued until information saturation was achieved. Data analysis followed Colaizzi’s seven-step approach: (1) reading all participants’ descriptions to gain a general understanding, (2) extracting significant statements, (3) formulating meanings from these statements, (4) organizing formulated meanings into clusters of themes, (5) developing an exhaustive description, (6) refining the description into a fundamental structure, and (7) validating the findings by returning to participants for feedback. A socialization theory framework was applied to analyze relationships between themes and clarify the mechanisms underlying professional identity development.

**Results:**

From March 1 to March 13, 2023, 21 nursing students from seven medical schools in Guangdong Province participated in the research. The findings showed that nursing students’ professional identity, initially medium or low before the epidemic, significantly improved afterward. Three key themes related to professional identity improvement were identified based on socialization theory: (1) Social Practice Experience: Activities such as volunteer services and pandemic prevention education promote role affirmation, meaningful engagement, and a strong professional mission. (2) Role Model Observation: Observing frontline healthcare workers enhances professional honor and role expectations. (3) Social Comparison and Evaluation: Positive feedback from media, family, and peers encourages respect and enthusiasm for nursing.

**Conclusion:**

The COVID-19 pandemic markedly improved nursing students’ professional identity, providing insights for education and career development. To strengthen nursing education, it is advised to emphasize disaster response training, integrate role model examples, and utilize social recognition to foster students’ professional identity and commitment.

## Introduction

Professional identity in nursing is defined as a nurse’s understanding and appraisal of their occupational role. This concept is deemed essential in both nursing education and practice (1). Nurses with a strong professional identity often demonstrate empathy, high-quality patient care, and effective teamwork (2). When nurses see their value and professional goals realized in their work, they are more likely to feel satisfied and remain in their roles long-term. Given the importance of professional identity in nursing, research suggests that fostering a nurse’s professional identity should start during their student years, supported by both educational and practical experiences (3).

Professional identity in nursing students refers to their positive perception of their chosen field during university. The strength of this identity impacts their career choices after graduation, the quality of clinical care, and the nursing workforce’s overall stability, quantity, and quality (4). International studies have shown that nursing students generally have a high level of professional identity. For example, research from Australia (5) and Turkey (6) reported similar findings. In South Korea, Koo et al. (7) reported that most nursing students exhibit moderate to high levels of professional identity. In China, nursing students often exhibit low professional identity, underscoring a critical challenge in education (8, 9). While direct comparisons between Chinese and international nursing students are limited, reviews indicate that global nursing education systems place greater emphasis on fostering professional identity. This is facilitated through clinical practice, career orientation, and planning, which aim to help students strengthen their commitment to the nursing profession (3, 10). Additionally, societal recognition, career development opportunities, and compensation significantly influence nursing students’ professional identity (8). The nursing profession’s societal esteem and career prospects strengthen students’ professional identity, fostering positivity and commitment to their careers.

COVID-19, a highly contagious respiratory disease, is caused by the novel coronavirus (11). During the pandemic, frontline nurses demonstrated expertise and dedication, collaborating with doctors to save lives, support recovery, and improve survival rates, gaining widespread recognition. The outbreak has increased societal recognition of nursing’s significance, subsequently raising nurses’ social status (12). In 2003, a qualitative study by Hong Kong scholars on nursing students during the SARS outbreak revealed a positive impact on their professional identity (13). Although studies on how the COVID-19 pandemic has influenced nursing students’ professional identity remain limited, evidence indicates notable trends. A study in Iran by Seyedeh et al. (14) surveyed all nursing students at Babol Medical College, revealing that internship nursing students exhibited improved professional identity during the pandemic. Similarly, Tang et al. (15) surveyed 3,875 undergraduate nursing students from seven Chinese universities between March and April 2020, reporting that professional identity strengthened during the later stages of the pandemic. In 2021, Zhang et al. (16) conducted a nationwide cross-sectional study and found that about 86.7% of Chinese nursing students believed the pandemic enhanced the image of nursing. Furthermore, greater psychological resilience is strongly correlated with a heightened professional identity.

Existing research, both domestic and international, indicates that the pandemic has strengthened nursing students’ professional identity. Nonetheless, most studies rely on quantitative methods, and the mechanisms through which the pandemic fosters this identity remain unclear. To address this gap, this study adopts a qualitative approach grounded in socialization theory. This approach explores how the pandemic shapes nursing students’ professional identity, providing insights to inform nursing education strategies aimed at strengthening it.

Socialization theory explains how professional identity is formed and evolves. It emphasizes that socialization is a dynamic and multifaceted process through which individuals acquire vocational knowledge and skills while developing a sense of understanding and identification with their roles via social interaction and comparison (17). Key components of this theory—self-enhancement, alternative reinforcement, and social comparison—help individuals build self-evaluation skills through practice, observe others to gain social experience, and refine their self-assessment through observation and comparison (18). Drawing on socialization theory and supporting literature, this study hypothesizes that the COVID-19 pandemic has impacted nursing students’ professional identity through these pathways: Firstly, students’ hands-on experiences during the pandemic may deepen their sense of purpose and commitment to the nursing profession (self-reinforcement). Secondly, observing frontline nursing staff as role models may inspire greater admiration and pride for the profession among students (vicarious reinforcement). Thirdly, societal recognition of nursing’s importance may boost students’ professional identity through social comparison. In summary, we propose that these mechanisms resulted in positive, significant changes in nursing students’ professional identity, fostering a stable and internalized professional transformation.

During the COVID-19 pandemic, nursing students experienced significant changes in their professional identity, yet the mechanisms driving these changes remain uncertain. This study applies socialization theory—focusing on ‘self-reinforcement,’ ‘vicarious reinforcement,’ and ‘social comparison’—to examine how pandemic-related social events shaped their professional identity. Using qualitative methods, the study demonstrates how experiential learning and role models enriched professional identity, offering guidance for post-pandemic nursing education and fresh perspectives for the field.

## Methods

### Design

This study employed the phenomenological method of qualitative research to explore nursing students’ personal experiences and emotions during professional socialization in the post-pandemic era through in-depth interviews and case studies. This method allowed the study to deeply examine nursing students’ personal experiences and emotions, providing a comprehensive understanding of their professional identity formation and evolution.

### Sampling and recruitment

Using purposive sampling, we recruited nursing students from seven medical colleges in Guangdong Province, China, between March 1 and March 13, 2023, as study participants. Inclusion criteria: Full-time nursing students (diploma, undergraduate, or graduate) aged 18–24, including those undergoing clinical internships, who provided informed consent. Exclusion criteria: Nursing students with more than 1 year of work experience, severe cognitive or mental disorders, or an inability to communicate in the research language.

### Sample size

The sample size for this study was determined by the requirements of qualitative research and data saturation principles. Researchers deemed the sample size sufficient when no new insights or themes emerged from the data.

### Data collection

The interview guide was developed through a systematic process. Initially, a research team member conducted a literature review spanning sociology, qualitative nursing research, and nursing education to draft a preliminary outline. The draft underwent internal review by three experienced clinical nurses with teaching expertise. External consultations were then conducted with a qualitative research expert and a nursing education expert. Pilot interviews with three nursing students, excluded from the final study sample, were conducted to refine the guide. The final guide focused on four key areas: (1) Actions taken leveraging nursing expertise during the pandemic; (2) Perceptions of the nursing profession before and after the pandemic; (3) Insights into frontline nursing work during the pandemic; (4) Post-pandemic perspectives on the nursing profession from those around you.

Before the interviews, researchers explained the study’s purpose and procedures, obtained signed informed consent, and assured participants of confidentiality using anonymized coding. Semi-structured interviews were conducted by two clinical nurses with over 10 years of experience and a senior nursing student in clinical training. All interviewers underwent standardized training in qualitative interviewing techniques to ensure consistency. Mechanisms to maintain consistency included: (1) a comprehensive interviewer manual; (2) routine peer reviews of interview recordings and transcripts; and (3) debriefing sessions among interviewers to align methods and analyze emerging patterns. Interviews were audio-recorded, with transcripts verified and prepared within 24 h to ensure accuracy.

### Data analysis

The data were analyzed using Colaizzi’s seven-step phenomenological method, ensuring rigor and a systematic approach. First, all transcripts were reviewed to gain an overall understanding of participants’ experiences. Key statements related to the phenomenon were extracted, and their meanings were organized into thematic clusters. A comprehensive description of the phenomenon was developed and then refined into a core structure representing shared experiences.

To ensure reliability and validity, several measures were implemented. Member checking involved returning findings to participants to confirm that the results accurately reflected their experiences. Two researchers independently coded and analyzed the data, resolving any discrepancies through discussion and consensus. A third qualitative research expert reviewed the final themes to ensure accuracy and reliability. Reflexive journals were used to document personal biases, assumptions, and decisions, ensuring reflexivity throughout the research process. An audit trail was implemented to ensure transparency during all stages of data collection and analysis. These measures collectively enhanced the findings’ trustworthiness, credibility, and authenticity.

### Ethical considerations

This study was conducted by the principles of the Declaration of Helsinki. The 471 study design was approved by the Standing Committee for Research Ethics at The 472 Sixth Affiliated Hospital, Sun Yat-sen University (2018ZSLYEC-036) in Guangzhou. The study protocol received approval from the Institutional Review Board. Participants were informed about the study’s objectives and methods and signed written informed consent. They were assured that participation was voluntary and that their data would stay confidential and would be used solely for research. Researchers assured participants could withdraw at any time without facing any consequences. All interview data, including recordings and transcripts, were securely stored and made accessible exclusively to the research team. To ensure anonymity, all personal information was replaced with coded identifiers instead of letters.

### Rigors

The study ensured rigor by utilizing Colaizzi’s descriptive phenomenological method and adhering to qualitative research principles for trustworthiness, credibility, and authenticity. Credibility was established through member checking, wherein participants validated the findings, and triangulation, which cross-verified data from multiple sources including interviews, field notes, and reflective journals. Dependability was ensured through independent coding and analysis by two researchers, with a third investigator reviewing the final themes for accuracy and reliability. Confirmability was achieved via reflexive journals, in which researchers recorded biases, assumptions, and decisions to maintain objectivity throughout the process. Finally, transparency was ensured through a comprehensive audit trail documenting all steps of the research process, enhancing replicability and reliability. Together, these measures strengthened the study’s rigor and reinforced the validity of its findings.

## Results

### Participants

The study included 21 participants aged 19 to 23, with 5 males and 16 females. The participants’ educational backgrounds included 2 from junior colleges, 12 from second-tier universities, and 7 from first-tier universities, all in Guangdong Province. Participants came from diverse provinces, including Guangdong (13), Xinjiang (1), Anhui (1), Gansu (1), Henan (1), and Guizhou (1). Participants’ academic levels included 1 sophomore, 5 juniors, 12 seniors, and 3 master’s students. Fourteen participants had completed clinical internships, while the other seven had not yet begun clinical practice. Twelve students had transferred to the nursing program during college admissions after being rejected from their first-choice programs. Six students planned to work in non-clinical nursing roles after graduation. Interview durations ranged from 18:22 to 46:41 min. Detailed demographic information is provided in Table 1.

**Table 1:** Demographic details of interviewees (*N* = 21).

ID	Gender	Age	Institution level	Year in program	Clinical internship	Native place	Major reallocation	Career intentions
P1	Female	22	Second-tier University	Senior	Yes	Shenzhen	Yes	Nurse
P2	Male	22	Second-tier University	Senior	Yes	Foshan	No	Nurse
P3	Male	21	Second-tier University	Junior	No	Huizhou	Yes	Nurse
P4	Female	20	Second-tier University	Sophomore	No	Guangzhou	No	Other
P5	Female	20	Junior College	Junior	Yes	Qingyuan	No	Nurse
P6	Female	21	Second-tier University	Junior	No	Shenzhen	No	Nurse
P7	Female	22	Second-tier University	Senior	Yes	Shenzhen	Yes	Nurse
P8	Female	23	Second-tier University	Senior	Yes	Zhanjiang	Yes	Entrepreneurship
P9	Male	22	First-tier University	Senior	Yes	Xinjiang	No	Nurse
P10	Female	22	Vocational College	Junior	Yes	Zhaoqing	Yes	Nurse
P11	Male	19	First-tier University	Junior	No	Anhui	Yes	Other
P12	Female	22	Second-tier University	Senior	Yes	Zhuhai	No	Nurse
P13	Female	22	Second-tier University	Senior	Yes	Shantou	No	Nurse
P14	Female	22	Second-tier University	Senior	Yes	Shenzhen	No	Other
P15	Female	22	First-tier University	Senior	Yes	Qingyuan	Yes	Nurse
P16	Male	22	Second-tier University	Senior	Yes	Guangzhou	No	Nurse
P17	Female	21	First-tier University	Senior	Yes	Qingyuan	Yes	Law
P18	Female	21	Second-tier University	Senior	Yes	Dongguan	Yes	Nurse
P19	Female	21	First-tier University	Master’s Year 1	Yes	Gansu	Yes	Teacher
P20	Female	23	First-tier University	Master’s Year 2	Yes	Henan	Yes	Nurse
P21	Female	23	First-tier University	Master’s Year 3	Yes	Guizhou	Yes	Nurse

### Pre-pandemic nursing students’ professional identity: moderate to low

Before the pandemic, nursing students generally exhibited a moderate to low professional identity, with six students explicitly stating their reluctance to pursue nursing as a career post-graduation. This reluctance stemmed from several factors: (1) some students perceived nursing as lacking technical depth, which diminished its professional value. For instance, P17 shared, “The initial college years were dedicated to basic medical knowledge. During my internship, it fell short of my expectations, lacking in technical complexity. The clinical tasks, including basic activities like making beds and washing hair, felt mundane and underutilized my education.” (2) The stress and physical demands of nursing were highlighted. P5 remarked, “Night shifts are exceedingly strenuous.” P12 noted, “Solo night shifts are concerning and feel perilous.” (3) Perceptions of inadequate compensation in nursing were common. P7 stated, “The disparity between the workload and income has led to a disfavor among my classmates and me toward nursing.” (4) The perceived low social status of nursing was also mentioned. P21 remarked, “Prejudices against our profession persist, diminishing its societal standing. There’s a misconception that our work is limited to menial tasks like changing bed sheets and attending to basic care needs.”

### Post-pandemic nursing students’ overall professional identity: significant improvement

The COVID-19 pandemic marked a turning point, significantly enhancing nursing students’ professional identity. Nursing was increasingly perceived as a noble and essential profession, fostering a deeper sense of mission, pride, and honor. Some students changed their career preferences as a result of the pandemic. P2: “Through this pandemic, I feel that nursing is truly great and important, and it has strengthened my determination to become an excellent clinical nurse.” P7: “This experience made me realize how important this profession is. In the face of diseases and disasters, medical personnel must always be on the front lines.” P9: “This pandemic has made me realize the greater importance of nursing work. My sense of professional identity has been enhanced as a result.” P12: “This pandemic made me realize that there is so much that can be done in nursing. I’ve started to feel a bit more inclined to go into clinical practice compared to before, and I now have more ideas about it.” P19: “After this pandemic, I have grown to admire and appreciate the nursing profession even more.” “Nurses played a crucial role during this pandemic and were an indispensable part of overcoming it.” P20: “Initially, I did not want to pursue nursing-related work, but this pandemic made me realize there is so much that can be achieved in nursing.”

### Mechanisms for the improvement of nursing students’ professional identity

**Figure 1:**
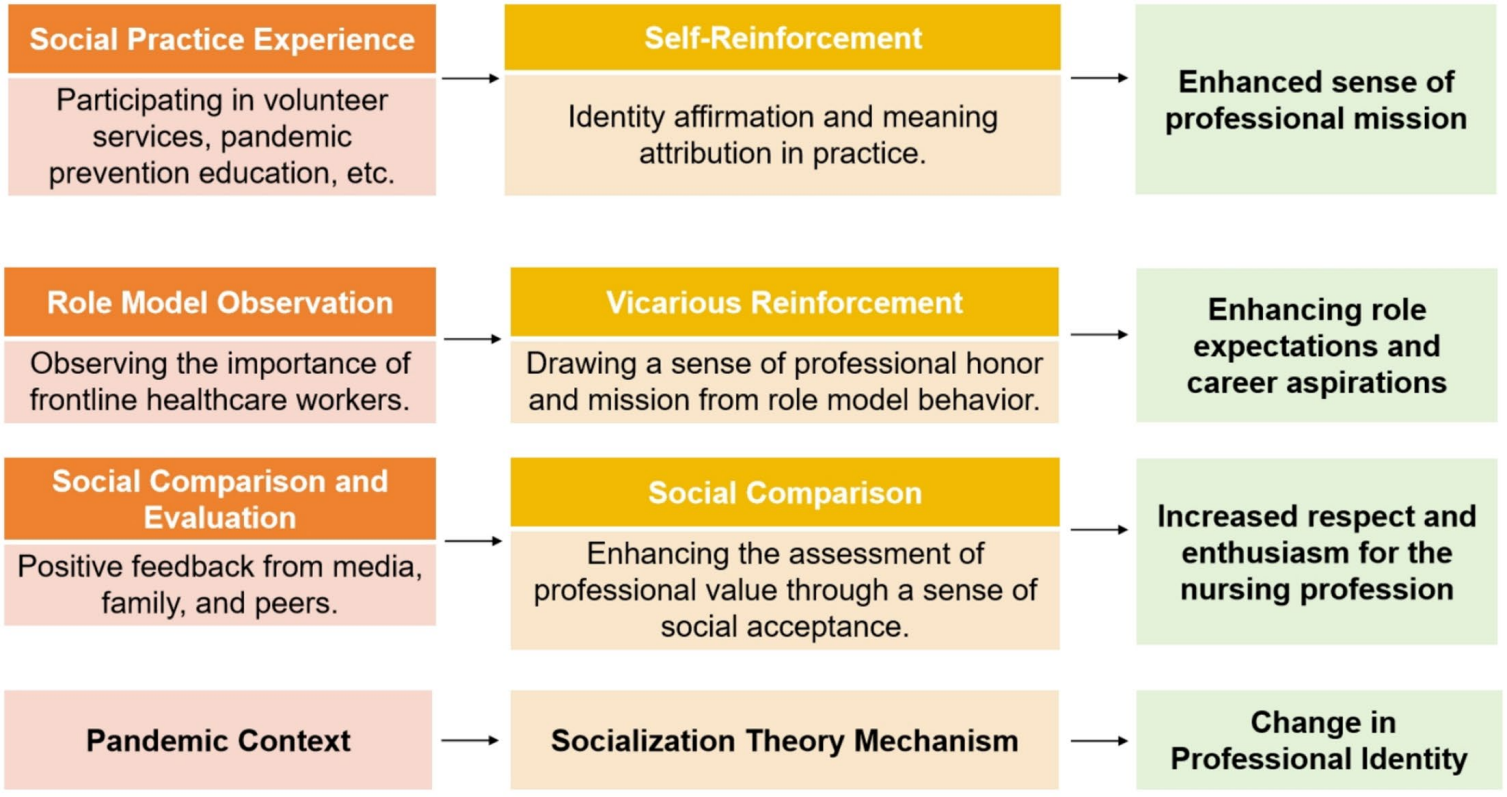
Socialization theory mechanism for enhancing nursing students’ professional identity.

According to socialization theory, the mechanisms for the improvement of nursing students’ professional identity include: (1) Social Practice Experience; (2) Role Model Observation; and (3) Social Comparison and Evaluation. Refer to Figure 1 for the detailed theoretical framework.

#### Social Practice Experience

Participating in volunteer services and pandemic prevention education affirmed students’ roles and provided meaning, enhancing their professional mission. Since the COVID-19 pandemic began, nursing students have actively applied their knowledge and skills to support the fight against the outbreak.

Two senior nursing students remained undeterred by the pandemic and actively volunteered in communities and villages. They applied their professional knowledge to grassroots prevention efforts, helping to contain the virus. They also trained non-medical volunteers to conduct health assessments accurately. P3 stated, “I volunteered at the village entrance to take temperatures and trained rural volunteers on using thermometers properly.” P10 remarked, “I worked with local organizations on temperature checks, traffic management, and crowd control.”

During the outbreak, numerous students encouraged their families and friends to minimize outings while actively demonstrating key protective measures such as wearing masks, washing hands, and disinfecting surfaces. They served as role models for their families and extended social circles. P7 shared, “I created handwashing videos and felt that spreading knowledge through these platforms was akin to sharing positive energy.” P14 stated, “I ensure that the older adult in my family wear masks and maintain hygiene. I also educate them on disease prevention by providing professional insights.” P18 elaborated, “I took the initiative to teach protective measures like the proper way to wear a mask, correcting common errors. Additionally, I introduced the concepts of contaminated, transitional, and clean zones to my family. They consult me for medical advice, reaffirming the importance of my nursing education.” P19 commented, “As a nursing student, my family views me as a trusted authority and often consults me regarding proper mask usage and handwashing techniques.” P21 expressed, “When the pandemic began, I advised my family to promptly buy masks, take necessary precautions, and make use of my professional expertise.”

Some students played a key role in ensuring social stability during the pandemic by sharing accurate information with their families and dispelling misinformation. P2 stated, “I share accurate information on my social media, such as WeChat and Weibo, adhering to a no-rumor policy. My friends trust my posts because of my nursing background.” P20 remarked, “During times of widespread rumors, people outside the medical field relied on me for trustworthy information.”

#### Role Model Observation

Observing frontline healthcare workers’ critical importance strengthened nursing students’ sense of professional honor and mission, enhancing their career aspirations. Amid the outbreak and lacking specific antiviral treatments, frontline nurses played a pivotal role in managing patient conditions, reducing mortality, and providing psychological care. P5: “In the cabin hospitals, I observed more psychological nursing, where nurses led exercises and offered emotional support. This experience underscored that nursing extends beyond procedures to embrace humanistic care.” P7: “I feel that nursing is truly remarkable. The senior colleagues on the frontlines are all exceptional. As the main force in combating the pandemic, nurses are exceptionally courageous. Observing how perilous and exhausting it is on the frontlines, I am deeply impressed that nurses are leading the charge.” P12: “News reports showed nurses wearing protective masks that left deep marks on their faces and avoiding hydration to reduce restroom breaks. Witnessing this, I felt nurses are truly heroic. This inspires me to become someone like them—a profession that offers immense fulfillment.” P15 commented, “Observing many teachers take the frontline evoked pride and strengthened my connection to nursing. The pandemic underscored the nobility and significance of nursing, confirming my desire to excel as a clinical nurse.” P16 stated, “Seeing teachers volunteer on the frontline was profoundly moving. Their dedication to saving lives and fighting the pandemic made me proud to be part of the nursing and healthcare community.”

When asked about participating in frontline duties during future public health crises, 13 students expressed their willingness, reflecting an increased sense of responsibility. P2 stated, “With my skills, I would undoubtedly join. Contributing to frontline work is my way of fulfilling my responsibilities.” P4 stated, “If needed and equipped with the required skills, I am ready to face any challenges on the frontline.” P7 affirmed, “I will go without hesitation! Taking on this responsibility, I am determined to actively serve on the frontline.” P15 remarked, “Choosing this profession, in my view, represents a commitment to taking on responsibility.”

#### Social Comparison and Evaluation

Through social comparison, positive feedback from the media, family, and peers significantly enhanced students’ respect for, and enthusiasm about, the nursing profession. P2: “Frontline medical workers save lives, and numerous positive reports have praised their actions. Healthcare workers in mainland China have demonstrated exceptional dedication.” P3: “The pandemic has provided an opportunity to highlight the role of medical staff. Many individuals shared positive stories on platforms like TikTok and Weibo, raising awareness of their critical importance.” P5: “Comments such as ‘I want to marry a nurse in the future’ are commonly seen on TikTok and Weibo, reflecting the elevated status of healthcare workers in society.” P11: “Numerous online reports commend healthcare workers. The pandemic has revealed their spirit of selfless dedication, fostering greater respect for medical professionals.” P19 stated, “My family became interested in hospital matters and actively discussed them with me.” P20 noted, “Experts emphasized the critical role of nurses during the pandemic. Afterward, I noticed progress in their career growth and societal recognition.” P21 commented, “I used to avoid questions about my major due to doubts about my career choice. Now, I proudly embrace it, as nursing is widely seen as noble.”

## Discussion

This is the first study employing qualitative research to examine changes in nursing students’ professional identity during the COVID-19 pandemic. Guided by socialization theory, the study investigates how “self-reinforcement,” “vicarious reinforcement,” and “social comparison” impact professional identity formation. The findings reveal that social events and practical experiences during the pandemic enhanced professional identity, providing critical insights for nursing education reform.

### Changes in nursing students’ professional identity

This study shows that before the pandemic, some nursing students exhibited moderate or low levels of professional identity, aligning with earlier research (8). Following the pandemic’s onset, their professional identity rose significantly, consistent with findings by Seyedeh et al. (8), Tang et al. (15), and Zhang et al. (16). This shift indicates that the pandemic was crucial in shaping professional socialization in nursing education.

### Mechanism analysis of the enhancement of nursing students’ professional identity

According to socialization theory, the pandemic strengthened nursing students’ professional identity through three mechanisms: self-reinforcement, vicarious reinforcement, and social comparison.

#### Self-reinforcement: identity confirmation and meaning in practice

During the pandemic, nursing students, like their peers in other countries (19, 20), engaged in volunteer work and epidemic prevention efforts, gaining valuable insights into the importance of nursing. These experiences fostered professional values and enhanced their sense of mission (21). Through patient care and prevention efforts, students recognized their contributions to society and patients, further solidifying their professional identity.

Positive practical experiences also boosted their confidence and sense of responsibility, inspiring enthusiasm for future work (19). Their strong performance during epidemic prevention underscored their social responsibility and professional commitment, both crucial for long-term careers in nursing (22).

#### Vicarious reinforcement: the demonstration effect of role model behavior

Vicarious reinforcement occurs when learning from observing others’ behaviors and experiences. During the pandemic, the role model behavior of frontline nurses was an important external factor in boosting nursing students’ professional identity. The pandemic enabled nursing students and nurses to witness their peers’ bravery and professionalism on the front lines, fostering admiration and deepening their identification with the profession. The widespread dissemination of nursing personnel’s dedication and sacrifices through social media and news showcased powerful professional role models. These events enhanced nursing students’ career aspirations and reinforced their recognition of the nursing profession’s value, bolstering their professional identity (23).

#### Social comparison: positive impact of rising social recognition on professional identity

Media coverage and public attention during the pandemic significantly enhanced the nursing profession’s social image (24). Nurses were celebrated as “anti-pandemic heroes,” elevating the profession’s status and instilling pride in nursing students. Support from families, schools, and society further fostered respect and enthusiasm for the profession, reinforcing students’ identity and passion.

### Implications for policy and practice

The COVID-19 pandemic has provided students with a unique opportunity for hands-on experiential learning. Research demonstrates that reflective learning centered on disaster events significantly enhances professional identity (25). The findings suggest three key recommendations for post-pandemic nursing education: (1) Strengthen Practical Learning: Utilize pandemic-related cases and simulations to immerse students in real-world scenarios while highlighting nursing’s critical value. (2) Leverage Role Models: Present frontline nurses’ stories through diverse mediums, such as lectures, interviews, and case studies, to inspire students and reinforce the profession’s significance. (3) Promote Social Recognition: Enhance the public image of nursing through media campaigns and supportive policies, incorporating this recognition into educational frameworks. Furthermore, the study emphasizes the need to strengthen disaster response capabilities and establish a robust disaster education system to prepare for future emergencies (26).

### Limitations

This study has several limitations. Firstly, the research involved 21 nursing students from seven medical schools in Guangdong Province, which limits the generalizability of findings. Future research with a larger, more geographically diverse participant pool is recommended to validate the results and provide a broader perspective. Secondly, data was collected after the peak of the COVID-19 pandemic (March 1 to March 13, 2023), making it difficult to directly capture pre-pandemic attitudes. Although retrospective questioning was used, reliance on self-reported data may introduce recall bias. Thirdly, the subjective nature of the phenomenological approach and semi-structured interviews may influence the findings, though double coding was used to enhance reliability. Lastly, the sample had a gender imbalance, reflecting nursing demographics, but limiting exploration of gender-specific differences. These limitations highlight areas for further exploration, which could provide additional insights into nursing students’ professional identity and its influencing factors.

## Conclusion

This study utilizes socialization theory to explore the core mechanisms that strengthen nursing students’ professional identity during the COVID-19 pandemic. It deepens our understanding of the impact of societal events on shaping professional identity. The findings offer both theoretical insights for nursing education and practical strategies for improving post-pandemic nursing talent development systems. These results have important implications for future nursing education strategies and workforce policies.

## Data Availability

The original contributions presented in the study are included in the article/supplementary material, further inquiries can be directed to the corresponding authors.
